# Improvements in Depression and Mental Health After Acceptance and Commitment Therapy are Related to Changes in Defusion and Values-Based Action

**DOI:** 10.1007/s10879-017-9367-6

**Published:** 2017-09-02

**Authors:** Kate Bramwell, Thomas Richardson

**Affiliations:** 1 Mental Health Recovery Team North, Solent NHS Trust, St. Mary’s Community Health Campus, Milton Road, Portsmouth, PO3 6AD UK; 2 School of Psychology, University of Southampton, Southampton, SO17 1BJ UK

**Keywords:** ACT, Processes, Fusion, Values, Distress, Depression

## Abstract

Acceptance and commitment therapy (ACT) has been found to be effective for various mental health disorders but the processes through which it affects change remain unclear. Much process research in the area is on physical rather than mental health, and focuses on the broad concept of psychological flexibility with little research on specific mechanisms identified in theory such as fusion and values. This study explored whether there was a relationship between two of the main ACT processes (cognitive defusion and values) and levels of depression and distress. Thirty-three participants completed questionnaires at the start and end of their treatment measuring general mental health and distress, depression, levels of cognitive fusion and how much they were living in line with their values and how important their values were to them. Results showed reductions in levels of fusion and increases in values-based action were significantly related to reductions in distress and depression. There was no correlation between changes in values importance and changes in distress or depression. This study therefore suggests decreased defusion and increased values-based action is an important mechanism in the efficacy of ACT in those with depression and mental health problems. The study is however limited by a small sample size and future research with a sample large enough for mediation analysis would be beneficial.

## Introduction

Psychological therapies have seen a shift in recent years from behavioural and cognitive behavioural treatments which emphasise changing the content of thoughts (Hollon and Beck 1994) to mindfulness-based, third wave treatments, characterised by a focus on metacognition, acceptance, mindfulness and the therapeutic relationship and emphasise changing the relationship with thoughts (Kahl et al. 2012). Acceptance and Commitment Therapy (ACT) has become one of the leading third-wave therapies in recent years in the treatment of physical and mental health difficulties (A-Tjak et al. 2015) and aims to improve functioning and quality of life by increasing psychological flexibility.

Psychological flexibility is defined as being able to act effectively, to live in line with one’s values, whilst accepting the presence of distressing or interfering thoughts, emotions and bodily sensations (Hayes et al. 2004). Hayes, Strosahl and Wilson (2011) conceptualise psychological flexibility as six overlapping processes: acceptance, cognitive defusion, being present, self-as-context, values orientation and committed action; each of these processes aims to increase psychological flexibility in different ways.

Hayes et al. (2004) describes the process of acceptance as aiming to increase “the active non-judgemental embracing of experience in the here and now” (p. 656). Cognitive defusion aims to help people notice their thoughts and feelings and relate to them differently (Hayes et al. 2004). Hayes et al. (2013) describe being present as “attending to what is present in a focused, voluntary, and flexible fashion, linked to one’s values and purposes” (p. 6) which enables people to experience the world more directly. Self-as-context aims to build awareness that a person’s fundamental self is unchanged by what is happening around them. This helps people to be aware of their own experiences without attaching to them, allowing a distinction to be made between the person having the experience and the experience itself (Hayes et al. 2013). In terms of values, ACT aims to help someone choose life directions in various domains e.g. family, career and helps people to move in the direction of those values whilst committed action involves engaging in behaviours in line with one’s values (Hayes et al. 2004). All of these processes have been found to affect treatment outcomes in different ways.

Studies have shown the process of cognitive defusion can be related to treatment outcomes; Zettle and Hayes (1986) and Zettle and Rains (1989) both found changes in cognitive defusion mediated treatment effects for ACT for depression when comparing ACT with Cognitive Therapy (CT) where the mediating effect was not present in the CT group. The extent to which people are living in line with their values has also been found to mediate treatment effects, for example, Lundgren et al. (2008) found in patients with epilepsy, values attainment had a mediating effect on pre to follow up changes on various outcome measures including quality of life and wellbeing after a course of ACT.

Research has shown these processes of psychological flexibility appear to account for a sizeable proportion of the benefits observed in ACT (McCracken and Gutiérrez-Martinez 2011) however many researchers agree further research is needed to clarify the roles each of the processes plays in change (McCracken and Gutiérrez-Martinez 2011; Wicksell et al. 2010). A recent meta-analysis concluded ACT is as effective as other established psychological interventions in treating anxiety disorders, depression and addiction (A-Tjak et al. 2015), however the reasons why ACT is effective are unknown. Furthermore, A-Tjak et al. (2015) suggest it is important to show ACT processes are critical to ACT outcomes as the vast majority of previous research has focused on whether improving psychological flexibility in general affects outcomes, rather than how specific processes affect outcomes.

Researchers are particularly interested in the change in ACT processes within treatment and how these changes relate to progress and improvement (Vowles et al. 2014). Although there is a small but growing body of literature exploring ACT processes, values-based action has been subject to less study than some of the other processes (Vowles et al. 2009) and researchers have suggested this is an area requiring further study (Forman et al. 2007). Furthermore, research has mainly examined values as a whole rather than values action and values importance, therefore this study will examine values importance and values action separately.

The study will also explore the process of cognitive fusion and it’s relationship to mental health and depression as researchers have suggested previously cited findings on fusion need to be replicated (Wicksell et al. 2010). It has also been suggested it is important to study ACT and it’s processes in standard outpatient clinical settings (Vowles et al. 2009) which this study will do. This will allow data to be collected from a naturalistic sample and therefore directly clinically relevant.

Furthermore, because most of the research on processes in ACT has been in the field of physical health, this study will aim to extend the existing literature on the processes of change in general mental health and depression. The study will focus on depressive symptoms in a largely depressed population given the current evidence suggesting ACT as an effective treatment for depression (A-Tjak et al. 2015); examining a population where ACT has been proven to be effective will allow exploration of the processes by which this happens.

Given previous findings, this study hypothesises:

Reductions in general mental health symptoms will be related to decreased in levels of fusion and increases in values importance and values based action.

Reductions in depression symptoms will be related to decreases in levels of fusion and increases in values importance and values based action.

## Method and Materials

### Design and Participants

The study employed a repeated measures design analysing secondary data from a pre-post open trial design (Richardson et al. 2017). The difference between this previous study and this current study is that the initial study simply examined efficacy of ACT in this context by examining whether changes on measures were significant over time. This current study is a process study which will look at whether these changes in mental health are related to changes in ACT mechanisms. This study was an evaluation of existing data collected as part of routine service evaluation of existing clinical practice.

Eight staff were qualified psychological therapists (clinical or counselling psychologists and one psychotherapist) and twelve were mental health nurses or social workers with one occupational therapist. All undertook 5 days ACT-specific training.

Participants were 33 service users who were receiving individual ACT in a community mental health team in the South of England. 72.7% (n = 24) of the sample were female and 27.3% (n = 9) of the sample were male. Ages ranged from 21 to 67 with a mean age of 42 and the sample was 93.9% White British.

Participants were from two pathways within the service (trans-diagnostic and depression pathways). 39.4% (n = 13) of participants had a primary diagnosis of depression, however participants with a range of diagnoses were included. 48.5% (n = 16) had a secondary diagnosis. Diagnosis was ascertained from participants’ medical notes.

There were no specific inclusion/ exclusion criteria for this research as this was a service evaluation in a clinical setting, providing useful practice-based evidence. The study used data from routine clinical practice: all those who were referred for therapy were invited to take part. Only participants who completed treatment were included in the analysis. Clinicians did not use a manual but all read the same book and used similar exercises from this (Harris 2009). Cases were supervised in regular group supervision. The mean number of sessions completed was 13 with a range from 4 to 24.

### Measures

*Patient Health Questionnaire (PHQ-9)* (Kroenke et al. 2001). This measured the severity of depression symptoms and is reliable and valid (Kroenke et al. 2001). Example questions include ‘Over the past 2 weeks, how often have you been bothered by feeling down, depressed or hopeless’ and participants must rate how often this is the case on a scale of 0 (not at all) to 3 (nearly every day) to receive a total score between 0 and 27. Cronbach’s alpha in this study was 0.74 compared to 0.89 in a primary care sample (Kroenke et al. 2001). Test–retest reliability of the PHQ-9 has also been found to be “excellent” (Kroenke et al. 2001).

*Clinical Outcomes in Routine Evaluation (CORE-OM)* (Evans et al. 2000). This measured general mental health and distress over the past week and is reliable and valid (Evans et al. 2002). Example questions include ‘Over the last week, I have felt tense, anxious or nervous’ and participants rate how often this is the case on a scale of 0 (not at all) to 4 (most or all of the time) to receive a total score between 0 and 136. The total score for all items was used in this study and Cronbach’s alpha in this study was 0.86 compared to 0.94 in a normative sample (Evans et al. 2002). The CORE-OM has high test–retest reliability (Evans et al. 2000).

*Valued Living Questionnaire (VLQ)* (Wilson et al. 2010). This measured how important particular values were to people and the extent to which people were living in line with these values. It has been shown to have good reliability (Wilson et al. 2010). Participants rate on scales of 1–10 how important ten domains of life are to them such as family or work (values importance score) and how consistently they are living in line with such values (values action score). The highest score for importance for each domain is 10 and the highest score for action for each domain is 10 to score a total importance score out of 100 and a total action score out of 100. In the current study, a total action score and a total importance score was used. Cronbach’s alpha was 0.75 for the action scale and 0.82 for the importance scale compared to 0.77 and 0.75 in the normative sample (Wilson et al. 2010). The VLQ has good test–retest reliability (Wilson et al. 2010).

*Cognitive Fusion Questionnaire (CFQ)* (Gillanders et al. 2014). This assessed fusion and included questions relating to the extent to which people’s thoughts are distressing and interfere with action. Example questions include ‘I struggle with my thoughts’ and participants must rate how true the statements are on a scale of 1 (never true) to 7 (always true). Participants receive a total score out of 49. This has been shown to be reliable and valid (Gillanders et al. 2014). Cronbach’s alpha in this study was 0.82 compared to 0.81 in the normative sample. The CFQ has good test–retest reliability (Gillanders et al. 2014).

### Procedure

Participants were invited by their therapist when they started treatment to complete the questionnaires and sign a consent form to take part in the study. Informed consent was obtained from all individual participants included in the study. Participants were asked by their therapist to complete the questionnaires again at the end of treatment. Treatment targeted the six key processes of ACT and was delivered by an ACT-trained therapist (mental health nurse, clinical psychologist or social worker).

### Statistical Analysis

Total scores were calculated using SPSS for PHQ-9, CFQ, VLQ action and importance and CORE totals. All change scores were calculated by subtracting post scores from pre scores. Statistics were not calculated in this study to determine whether changes were significant, however this data has been used in a previous service evaluation where pre to post changes were found to be statistically significant for all measures (Richardson et al. 2017). There was insufficient sample size for a regression. Bivariate Pearson’s correlations were therefore conducted with PHQ-9 change scores and CFQ, VLQ action and importance change scores. Bivariate Pearson’s correlations were also conducted with CORE total change scores and CFQ, VLQ action and importance change scores.

Missing data was substituted with the mean value as data was deemed to be missing at random and mean substitution is a reasonable estimate for observations missing from a normal distribution (Kang 2013) as was the case with this data set. Furthermore, the missing rate of response was relatively low: with missing items pre therapy for two individuals for the CORE total, one for the PHQ total and one for the CFQ total. Missing data was slightly higher for VLQ importance (four individuals) and VLQ action totals (five individuals).Thus the limitations of mean substitutions seemed minimal given the low level of missing data. Only participants who completed treatment were included in the analysis.

## Results

Results from all 33 participants were included in the analysis. 72.7% (n = 24) of the sample were female and 27.3% (n = 9) of the sample were male. Ages ranged from 21 to 67 with a mean age of 42 and the sample was 93.9% White British. Descriptive statistics were calculated for pre, post and change scores on all measures and can be seen in Table 1.

**Table 1:** Descriptive statistics for pre, post and change scores for all measures

Measure	Pre	Post	Change
	*M (SD)*	*M (SD)*	*M (SD)*
PHQ-9	18.3 (5)	11.49 (6.47)	−6.82 (7.22)
CORE	77.4 (15.42)	50.67 (26.7)	−26.74 (26.74)
CFQ	41.82 (4.52)	30.03 (9.07)	−11.79 (10.17)
VLQ importance	58.94 (20.97)	63.53 (18.94)	4.59 (10.62)
VLQ action	34.47 (14.26)	45.91 (16.32)	11.44 (14.05)

*N* = 33

### Bivariate Correlations

As seen in Table 2, significant positive correlations were found between PHQ-9 change scores and CFQ change scores indicating a relationship between reductions in levels of depression and reductions in levels of cognitive fusion. Significant negative correlations were found between PHQ-9 change scores and VLQ action change scores indicating a relationship between reductions in levels of depression and increases in values action. There were no significant correlations between PHQ-9 change scores and VLQ importance change scores.

**Table 2:** Correlations between change scores on all measures

Measure		PHQ-9	CFQ	VLQ action	VLQ importance	CORE
PHQ-9	*r*	1	.575*	−.504*	−.158	.86*
	*p*		.000	.003	.38	.000
CFQ	*r*	.575*	1	−.229	.028	.601*
	*p*	.000		.2	.878	.000
VLQ action	*r*	−.504*	−.229	1	.508*	−.555*
	*p*	.003	.2		.003	.001
VLQ importance	*r*	−.158	.028	.508*	1	−.245
	*p*	.38	.878	.003		.169
CORE	*r*	.86*	.601*	−.555*	−.245	1
	*p*	.000	.000	.001	.169	

*indicates significant correlation

As seen in Table 2, significant positive correlations were found between total CORE change scores and CFQ change scores indicating a relationship between reductions in levels of distress and reductions in levels of cognitive fusion. Significant negative correlations were found between total CORE change scores and VLQ action change scores indicating a relationship between reductions in levels of distress and increases in values action. There were no significant correlations between total CORE change scores and VLQ importance change scores.

## Discussion

This study showed there was a significant relationship between reductions in levels of fusion and reductions in distress and depression. There was also a significant relationship between increases in values-based action and reductions in distress and depression. These findings were consistent with the hypotheses and the current literature, for example this study supported previous findings by Vowles and McCracken (2008) that increases in values based action were significantly related to lower levels of distress and depression in a population of people with chronic pain. This study extended this finding by showing this relationship in a mental health context. Previous studies have also found changes in levels of cognitive fusion to be related to improved treatment outcomes after ACT for depression and psychosis (Bach and Hayes 2002; Gaudiano and Herbert 2006) and this study supported this finding. This was an important finding as researchers have said the previous results need to be replicated to add to the research on ACT processes and the mechanisms of change (Wicksell et al. 2010). Furthermore, given there was no correlation between CFQ and VLQ action or importance change scores, this suggests these are two distinct processes, lending further support to research suggesting different ACT processes affect outcomes in different ways.

It is of note however that previous research suggests improvements in depressive symptoms are related to improvements in one’s self concept and sense of self (Hayes et al. 2005). It is therefore possible that the changes in fusion and values could result, to an extent, from symptom reduction rather than them playing a part in leading to symptom reduction. The research design of this paper does not allow to prove or disprove this as we were unable to demonstrate a causal relationship; this is something future research could explore. However, Gloster et al. (2017) suggested increases in values action preceded decreases in suffering and therefore suggests the changes in values may happen before symptom reduction, lending support to our hypothesis.

The study also showed there was no correlation between changes in values importance and changes in distress or depression, which was unexpected and not consistent with the study’s hypotheses. This may be because the small amount of previous research exploring the process of values in ACT has either mainly looked at whether values-based action is related to treatment outcomes or uses values as a whole without differentiating between values-based action and values importance (e.g. Lundgren et al. 2008). Furthermore, values interventions in ACT tend to direct therapy by targeting things that get in the way of people living a valued life (Hayes et al. 2004) and it may be that values work tends to focus on ways to move around the barriers, ie committed action. Therefore perhaps values clarification and reflections on how important they are, formed a very small part of the treatment in comparison to work on defusion and values-based action. This is supported by recent research on the temporal process of change in ACT which suggests that values importance is initiated at the beginning of therapy before going on to focus on values action (Gloster et al. 2017) suggesting perhaps more time is spent on values action than values importance. The current study added to previous studies on values by explicitly looking at values importance and values action separately.

The study sought to replicate previous findings that changes in levels of cognitive fusion are related to changes in mental health and hoped to add to the gap in the literature on ACT processes by exploring the relationship between values-based action, values importance and mental health. It helps support previous research regarding fusion and values-based action and provided novel findings on values importance. The study is ecologically valid as it was an existing groups sample with no exclusion criteria and therefore provides useful information for real-life clinical settings. The repeated measures design also reduced variability in the results and helped improve their validity. The study provided useful information for clinicians practising ACT as knowing cognitive defusion and values-based action can influence treatment outcomes could help them to focus treatment accordingly. The results may also help clinicians in their use of clinical supervision to discuss ways to focus on particularly important processes. This in turn may benefit service users if treatment is focused appropriately for them and clinicians feel confident in doing this.

In terms of limitations, the study did not exclude participants on the basis of diagnosis, it should be noted a large proportion of the sample had a primary diagnosis of depression and therefore the results are perhaps more applicable to depression than other disorders. Furthermore, the experience of the therapists delivering the therapy was unknown and treatment did not follow a protocol; this may have led to large variations within treatment of what was covered and how well delivered the therapy was. There was also great variation in the number of sessions people received which may have meant some people included in the analysis received very little treatment focusing on values or fusion. Furthermore, this study could only examine outcomes of completers as it was looking at correlates of changes from therapy and post measures were only given for completers. However, it is important to note that in this sample, those who dropped out of therapy had lower depression and fusion scores (Richardson et al. 2017), thus this may have impacted results. Finally, the study was correlational in nature and thus causation cannot be inferred from the results.

In future studies, it would be useful to collect enough data to be able to conduct a mediation analysis or multiple regression to determine whether values action and importance and levels of fusion mediate changes in mental health. It may also be useful to explore relationships between change scores in the CORE subscales and changes in fusion and values-based action to understand what it is that changes in fusion and changes in values-based action is specifically improving in terms of general mental health.

This paper has shown reductions in levels of fusion and increases in values-based action are related to improvements in depressive symptoms and general mental health. This provides support for previous studies showing cognitive fusion to be related to improvements in depression but was an important finding as this was the first study to show this in routine outpatient clinical setting with a naturalistic sample. The study provided novel findings in terms of values as it separated values importance and values action where previous studies have explored values as a whole. The study added to the literature on processes in ACT, providing novel findings in the field of mental health and provides a solid basis for future research to explore more explicitly how these processes affect change in ACT.
